# Socio-demographic factors and the quality of life of those involved in the hostilities in Ukraine

**DOI:** 10.1186/s12955-026-02604-x

**Published:** 2026-08-07

**Authors:** Stanisław Fel, Krzysztof Jurek, Jarosław Kozak

**Affiliations:** Institute of Sociological Sciences, The John Paul II Catholic University of Lublin, Lublin, Poland

**Keywords:** Quality of life, Socio-demographic factors, War, Civilian population, Ukraine, WHOQOL-BREF, QOL predictors

## Abstract

The ongoing war in Ukraine has led to mass migrations of people, a destabilization of social institutions, and restricted access to basic life resources, which undermines the conditions for maintaining quality of life (QOL). The paper aims to identify the determinants QOL in four domains: physical health, psychological health, social relationships, and environment. The survey was carried out in 2019 amongst 314 adults displaced from the Donbas and living in central and western Ukraine. We used quantitative measures based on the WHOQOL-BREF questionnaire and performed multiple regression analysis. The regression analysis revealed that savings significantly predicted QOL in three domains (physical, psychological, and environment, whereas self-perceived material security played a key role in the domain social relationships domain. Other significant predictors were gender, place of residence, and distance from hostilities (for physical health), continuation of education (for environment), and health insurance (for psychological health and environment). The results suggest the need to redefine humanitarian support towards actions enabling not only survival but also the maintenance of social and psychological functioning. Special attention should be devoted to enhancing cross-domain resources (e.g., savings), access to institutional structures (education, insurance), and subjective sense of security as protective factors for QOL in wartime conditions.

## Introduction

The armed conflict in Ukraine has led to one of the greatest contemporary humanitarian catastrophes in Europe, with an impact on direct participants in hostilities [31]. The psychological, health, material, and social burdens resulting from war and forced displacements pose a serious threat to quality of life (QOL) among the victims of the conflict [30]. In the face of the long-term effects of war, understanding the determinants of QOL becomes not only a scientific challenge but also a social priority.

In this context, QOL is a useful research category, enabling a multifaceted assessment of individuals’ functioning in crisis conditions [48]. This perspective takes into account not only physical and psychological health but also social relationships and the perception of the living environment [12]. An analysis of QOL can, therefore, provide data supporting the design of interventions aimed at improving the population’s well-being in situations of prolonged threat.

### Literature review

One of the dimensions of QOL most often analyzed in the context of war is psychological health [20]. Research conducted among the inhabitants of Ukraine a year after the full-scale invasion revealed that symptoms of depression, anxiety, Post-Traumatic Stress Disorder (PTSD), and complex PTSD (CPTSD) were significantly related to lowered QOL [26, 53]. Particularly strong relationships were observed between the number of traumatic events experienced and the level of depressive and anxiety symptoms [44]. Loneliness and a subjective sense of lack of meaning and control additionally intensify the decrease in psychological well-being and in overall life assessment [27]. Prolonged exposure to war trauma, even after the end of hostilities, can lead to chronic decrease in QOL [37], and depressive symptoms are considered to be the strongest individual factor associated with a decline in well-being [22].

Research conducted during the war in Ukraine revealed a significant deterioration of physical health indicators among the Ukrainian population, intensified by factors such as losses in employment, finance, and housing caused by the war [29]. It was found that after the outbreak of the war Ukrainians more frequently reported sleeping problems and panic attacks; there was also an increase in the prevalence of acute diseases of the respiratory system and chronic diseases [52]. As found, for example, in research among individuals suffering from epilepsy in Ukraine, during armed conflict, 52.0% of patients experienced an intensification of epileptic fits [17].

An equally important dimension of QOL among participants in hostilities is social relationships. Strong family relationships, a sense of belonging to the community, and access to social support are protective factors that can significantly mitigate the effects of wartime and migratory stressors [15, 21]. Among the refugees from Ukraine residing in Germany, it was observed that individuals who had social support reported higher QOL than lonely ones, despite a similar level of exposure to trauma [11]. Social isolation, including the language and cultural barrier, is a significant predictor of lowered well-being among people affected by forced migration and the experience of war [2, 3]. Research also showed that individuals using adaptive strategies, such as support seeking, positive reinterpretation, or planning, had higher QOL than those using maladaptive strategies, such as avoidance, withdrawal, or denial [53].

The environment, understood as the physical, material, and occupational setting that shapes people’s everyday wartime experience, may be related to QOL. Existing studies have shown that employment, even temporary, not only increases the sense of material security but also favors rebuilding a sense of agency and personal identity [13]. Lack of access to health services and medicines, especially in individuals with chronic diseases, dramatically lowers QOL during hostilities [20]. The results of research conducted among participants in hostilities in Ukraine additionally indicate that continuation of education, particularly when combined with professional psychological support, may alleviate depressive symptoms not only in university students but also in older adults in conditions of high exposure to trauma [19, 42].

The studies cited demonstrate the multifaceted consequences of war for quality of life. The data used in this article were collected in 2019 and therefore relate to an earlier phase of the war, linked to the armed conflict in the Donbas following 2014. For this reason, the findings should be interpreted primarily in the context of the long-term effects of the armed conflict, forced displacement and life under the chronic threat of war escalation.

### Aim

The present study is based on the assumption that QOL in extreme situations, such as war, should be analyzed as a multidimensional construct, sensitive to diverse psychosocial, material, and environmental factors. In the WHOQOL-BREF model [1], QOL is understood as the subjective assessment of well-being in four domains: physical health, psychological health, social relationships, and environment [12]. Physical health refers to the levels of energy, perceived pain, and everyday functioning capacity [14]. Psychological health encompasses emotional well-being, stress level, self-image, and sense of meaning in life [7]. Social relationships refer to closeness, support, and the quality of interactions with others [28]. Environment comprises housing conditions, security, and access to services as well as local and institutional resources [16]. In the present study, we assumed that each of the four domains of QOL reflected a different type of relationship between an individual’s experience and their demographic situation and access to specific resources, such as savings, insurance, education, or sense of material security [24].

Individuals strive to acquire, protect and restore the resources necessary for their functioning, whilst stress arises when these resources are lost, threatened or prove insufficient [26]. In wartime, the loss of resources is multidimensional. It encompasses not only health and physical safety, but also material stability, access to institutions, the ability to continue one’s education, social relationships and a sense of control over one’s own life [23]. From this perspective, the socio-demographic factors analysed in the study can be regarded not merely as descriptive characteristics of the sample, but as indicators of access to resources that safeguard quality of life in a situation of chronic threat.

The aim of the study was to identify the factors associated with QOL among participants in hostilities remaining in the territory of Ukraine during the ongoing armed conflict. As mentioned before, the analysis included four core dimensions of QOL, in accordance with the WHOQOL-BREF: physical health, psychological health, social relationships, and environment. The study aimed to determine to what extent sociodemographic factors, such as age, gender, place of residence, distance from hostilities, material situation, access to health insurance, education level, and savings, were related to the level of QOL in the four dimensions listed above in conditions of chronic threat.

The sociological character of the study, focused on QOL among participants in the war in Ukraine allows for analyzing the impact of sociodemographic factors on their everyday functioning in the conditions of the ongoing conflict. Filling this research gap can provide not only significant scientific data but also knowledge necessary for designing effective multidimensional assistance interventions, suited to the varied needs of individuals affected by armed conflicts.

## Materials and methods

### Design

Data was collected through a cross-sectional survey utilizing a convenience sampling approach. Participants included adult Ukrainians from the Donbas and individuals displaced from the Donbass residing in central or western Ukraine. To safeguard both respondents and researchers, given the study’s location in conflict zones, a non-probability snowball sampling method was employed for recruitment. The research was conducted in 2019 at hospitals, readaptation centers, and other locations where displaced persons were living.

Participants were informed about the objectives of the study, and their involvement was both anonymous and voluntary. The research was carried out by trained interviewers from Ukraine and supervised by researchers from the Institute of Sociological Sciences of the John Paul II Catholic University of Lublin, Poland. The data collection involved face-to-face interviews conducted in Ukrainian, during which participants completed a questionnaire package. The data collected were reviewed and verified for completeness prior to entry, with incomplete questionnaires being discarded.

###  Statistical analysis

Statistical analysis was carried out with IBM SPSS Statistics, version 29. Stepwise multiple linear regression analysis was used to identify the variables that predicted domain scores on the WHOQOL-BREF (with the variables shown in Table 1 included in the model). To assess multicollinearity, we computed the variance inflation factor (VIF). The values of all variables were in the acceptable range. In addition, the assumptions of linearity and homogeneity of variance were checked using scatter plots.

The level of significance was set at *p* < 0.05.

### Ethical approval

The study was conducted in accordance with the Declaration of Helsinki and was approved by the Research Ethics Committee of the Institute of Sociological Sciences at the John Paul II Catholic University of Lublin, Poland (approval No. KEB-IS-3/2019). Informed consent was obtained from all individual participants included in the study.

### Participants

The study involved 314 participants. Their mean age was 34.08 years (SD = 9.83, range 18–74).

**Table 1:** Sample characteristics (*N* = 314)

Variable		*n*	%
Age	≤ 25	62	19.7%
	26–30	63	20.1%
	31–35	62	19.7%
	36–40	43	13.7%
	41–45	29	9.2%
	46–50	27	8.6%
	> 50	17	5.4%
	N/A	11	3.5%
Gender	Female	74	23.6%
	Male	232	73.9%
	N/A	8	2.5%
Civil status	Married/cohabiting	127	40.4%
	Single/separated/divorced/widowed	172	54.8%
	N/A	15	4.8%
Children	Yes	192	61.1%
	No	116	36.9%
	N/A	6	1.9%
Education	Elementary/secondary	165	52.5%
	Higher	132	42.0%
	N/A	17	5.4%
Continuation of education	Yes	68	21.7%
	No	240	76.4%
	N/A	6	1.9%
Place of residence	Village	67	21.3%
	City	236	75.2%
	N/A	11	3.5%
Material status	Poor	144	45.9%
	Good	163	51.9%
	N/A	7	2.2%
Loss of a loved one	Yes	57	18.2%
	No	245	78.0%
	N/A	12	3.8%
Distance from hostilities	≤ 500 km	71	22.6%
	501–999 km	171	54.5%
	≥ 1000 km	41	13.1%
	N/A	31	9.9%
Health insurance	Yes	54	17.2%
	No	255	81.2%
	N/A	5	1.6%
Savings	Yes	75	23.9%
	No	233	74.2%
	N/A	6	1.9%
Material security	Yes	145	46.2%
	No	163	51.9%
	N/A	6	1.9%

### Variables and instruments

The following variables and instruments were included in the assessment:

#### Sociodemographic variables

The interview included a form for collecting sociodemographic data: age, gender, civil status, children, education, continuation of education, place of residence, loss of a loved one, distance from hostilities, health insurance, savings, and material security.

#### Quality of life

A reduced version of the World Health Organization Quality of Life BREF scale (WHOQOL-BREF-26) was used to assess quality of life. It consists of eight items – questions with five Likert-type answer options, asking the subject about different aspects of their QOL during the past month; it also includes two additional global questions: “How would you rate your quality of life?” and “How satisfied are you with your health?” The WHOQOL-BREF is a 26-item instrument covering four domains: physical health (7 items), psychological health (6 items), social relationships (3 items), and environment(8 items) (Table 2).

**Table 2:** Descriptive statistics for QOL domains [WHOQOL-BREF]

	M	Me	SD	A	K	Min.	Max.
Physical Health Domain	13.35	13.14	2.23	0.00	-0.26	7.43	18.86
Psychological Health Domain	12.83	12.67	2.18	-0.07	0.42	4.00	18.67
Social Relationships Domain	14.14	14.67	2.74	-0.53	0.57	4.00	20.00
Environment Domain	11.95	12.00	2.44	0.18	-0.01	5.00	19.00

M – mean; SD - standard deviation; Me – median; A – skewness; K – kurtosis, Min - minimum value; Max – maximum value

## Results

### Predicting the physical health domain of QOL

Table 3 shows multiple regression analyses predicting the physical health domain of QOL (final model). The model included five significant predictors: age (β = -0.294, t = -3.772, *p* < 0.001), gender (β = 0.307, t = 4.534, *p* = 0.012), distance from hostilities, (β = 0.131, t = 2.085, *p* = 0.038), place of residence (β = 0.148, t = 2.445, *p* = 0.015), and savings (β = 0.133, t = 2.065, *p* = 0.040). The final model (F(13, 217) = 7.665, *p* < 0.001) predicted 27% of the variance in the dependent variable (adj. R^2^ = 0.274). QOL was higher in younger respondents, women, individuals living farther away from areas where hostilities were taking place, those living in villages, and those who had savings.

**Table 3:** Multiple regression analysis predicting the physical health domain of QOL (final model)

	Unstandardized coefficients		Beta	t	*p*	95% CI		Collinearity	
	B	SE				Lower	Upper	Tol.	VIF
Constant	13.909	0.838		16.596	< 0.001	12.258	15.561		
Age	-0.068	0.018	-0.294	-3.772	**< 0.001**	-0.103	-0.032	0.519	1.928
Gender	1.588	0.350	0.307	4.534	**< 0.001**	0.898	2.279	0.690	1.450
Civil status	-0.181	0.361	-0.039	-0.501	0.617	-0.893	0.531	0.528	1.895
Children	-0.601	0.41	-0.127	-1.465	0.144	-1.408	0.207	0.421	2.376
Loss of a loved one	-0.487	0.353	-0.083	-1.38	0.169	-1.182	0.208	0.873	1.145
Education	-0.099	0.278	-0.022	-0.357	0.721	-0.648	0.449	0.854	1.171
Continuation of education	-0.091	0.416	-0.016	-0.219	0.827	-0.911	0.729	0.596	1.678
Distance from hostilities	0.496	0.238	0.131	2.085	**0.038**	0.027	0.966	0.804	1.243
Place of residence	0.850	0.348	0.148	2.445	**0.015**	0.165	1.536	0.867	1.153
Material status	0.405	0.285	0.089	1.423	0.156	-0.156	0.966	0.813	1.231
Health insurance	0.117	0.39	0.020	0.299	0.765	-0.652	0.885	0.741	1.350
Savings	0.718	0.348	0.133	2.065	**0.040**	0.033	1.404	0.759	1.317
Material security	-0.112	0.301	-0.024	-0.372	0.710	-0.706	0.482	0.735	1.361

B – estimated regression coefficient; SE – standard error; beta - standardized regression coefficient; t- t-statistic (for testing coefficient); p - p-value (significance level); 95% CI − 95% confidence interval; tol. - tolerance (measure of multicollinearity); VIF - variance inflation factor (multicollinearity indicator)

### Predicting the psychological health domain of QOL

Table 4 shows multiple regression analyses predicting the psychological health domain of QOL (final model). The model included two significant predictors: health insurance (β = 0.150, t = 2.039, *p* = 0.043) and savings (β = 0.147, t = 2.020, *p* = 0.045). The final model (F(13, 219) = 2.324, *p* = 0.007) predicted 7% of the variance in the dependent variable (adj. R^2^ = 0.069). Higher QOL was associated with having savings and having health insurance.

**Table 4:** Multiple regression analysis predicting the psychological health domain of QOL (final model)

	Unstandardized coefficients		Beta	t	*p*	95% CI		Collinearity	
	B	SE				Lower	Upper	Tol.	VIF
Constant	12.784	0.904		14.147	< 0.001	11.003	14.565		
Age	-0.036	0.020	-0.161	-1.817	0.071	-0.074	0.003	0.513	1.949
Gender	0.024	0.381	0.005	0.063	0.949	-0.727	0.775	0.688	1.454
Civil status	-0.359	0.395	-0.080	-0.908	0.365	-1.137	0.420	0.522	1.916
Children	-0.256	0.447	-0.056	-0.572	0.568	-1.137	0.626	0.417	2.398
Loss of a loved one	0.382	0.385	0.067	0.993	0.322	-0.377	1.141	0.874	1.144
Education	0.239	0.303	0.054	0.789	0.431	-0.358	0.836	0.857	1.167
Continuation of education	0.498	0.450	0.091	1.108	0.269	-0.388	1.385	0.599	1.669
Distance from hostilities	0.424	0.254	0.116	1.667	0.097	-0.077	0.925	0.824	1.213
Place of residence	0.483	0.377	0.087	1.281	0.201	-0.260	1.226	0.867	1.153
Material status	-0.449	0.309	-0.102	-1.455	0.147	-1.058	0.159	0.818	1.222
Health insurance	0.863	0.423	0.150	2.039	**0.043**	0.029	1.698	0.737	1.358
Savings	0.768	0.380	0.147	2.020	**0.045**	0.019	1.516	0.759	1.317
Material security	0.154	0.328	0.035	0.470	0.639	-0.492	0.800	0.737	1.356

B – estimated regression coefficient; SE – standard error; beta - standardized regression coefficient; t- t-statistic (for testing coefficient); p - p-value (significance level); 95% CI − 95% confidence interval; tol. - tolerance (measure of multicollinearity); VIF - variance inflation factor (multicollinearity indicator)

The study utilised the official Ukrainian version of the WHOQOL-BREF questionnaire provided by the World Health Organisation (WHO) (https://www.who.int/tools/whoqol/whoqol-bref/docs/default-source/publishing-policies/whoqol-bref/ukrainian-whoqol-bref). In order to assess the psychometric properties of the instrument in the study sample, an analysis of the internal consistency of the individual domains was carried out using Cronbach’s α. The values obtained were: 0.729 for the physical health domain, 0.741 for the mental health domain, 0.603 for the social relationships domain and 0.812 for the environment domain. The results indicate satisfactory internal consistency of the tool used, although the lower coefficient value for the social relationships domain is due to the fact that this domain comprises only three items.

### Predicting the social relationships domain of QOL

Table 5 shows multiple regression analyses predicting the social relationships domain of QOL (final model). The model included two significant predictors: age (β = -0.241, t = -2.706, *p* = 0.007) and material security (β = 0.154, t = 2.065, *p* = 0.040). The final model (F(13, 217) = 2.073, *p* = 0.017) predicted 5% of the variance in the dependent variable (adj. R^2^ = 0.057). Higher QOL was associated associated with lower age and material security.

**Table 5:** Multiple regression analysis predicting the social relationships domain of QOL (final model)

	Unstandardized coefficients		Beta	t	*p*	95% CI		Collinearity	
	B	SE				Lower	Upper	Tol.	VIF
Constant	15.360	1.149		13.366	0.000	13.095	17.625		
Age	-0.066	0.025	-0.241	-2.706	**0.007**	-0.115	-0.018	0.519	1.928
Gender	0.168	0.480	0.027	0.349	0.727	-0.779	1.114	0.690	1.450
Civil status	-0.850	0.495	-0.151	-1.717	0.087	-1.827	0.126	0.528	1.895
Children	-0.113	0.562	-0.020	-0.201	0.841	-1.221	0.995	0.421	2.376
Loss of a loved one	0.019	0.483	0.003	0.040	0.968	-0.934	0.972	0.873	1.145
Education	0.254	0.382	0.046	0.665	0.507	-0.498	1.006	0.854	1.171
Continuation of education	0.065	0.571	0.010	0.115	0.909	-1.059	1.190	0.596	1.678
Distance from hostilities	0.213	0.326	0.047	0.654	0.514	-0.430	0.857	0.804	1.243
Place of residence	0.595	0.477	0.086	1.249	0.213	-0.344	1.535	0.867	1.153
Material status	-0.130	0.390	-0.024	-0.333	0.740	-0.899	0.640	0.813	1.231
Health insurance	1.013	0.535	0.141	1.894	0.060	-0.041	2.067	0.741	1.350
Savings	0.165	0.477	0.025	0.346	0.729	-0.775	1.105	0.759	1.317
Material security	0.853	0.413	0.154	2.065	**0.040**	0.039	1.668	0.735	1.361

B – estimated regression coefficient; SE – standard error; beta - standardized regression coefficient; t- t-statistic (for testing coefficient); p - p-value (significance level); 95% CI − 95% confidence interval; tol. - tolerance (measure of multicollinearity); VIF - variance inflation factor (multicollinearity indicator)

### Predicting the environmental domain of QOL

Table 6 shows multiple regression analyses predicting the environmental domain of QOL (final model). The model included three significant predictors: continuation of education (β = 0.200, t = 2.587, *p* = 0.010), health insurance (β = 0.233, t = 3.364, *p* = 0.001), and savings (β = 0.187, t = 2.726, *p* = 0.007). The final model (F(13, 217) = 4.907, *p* < 0.001) predicted 18% of the variance **in the dependent variable** (adj. R^2^ = 0.181). Higher QOL was associated with continuation of education, having health insurance, and having savings.

**Table 6:** Multiple regression analysis predicting the environmental domain of QOL (final model)

	Unstandardized coefficients		Beta		t		*p*		95% CI			Collinearity		
	B	SE							Lower		Upper	Tol.		VIF
Constant	11.528	0.962			11.980		0.000		9.631		13.424			
Age	-0.023	0.021	-0.093		-1.127		0.261		-0.064		0.017	0.519		1.928
Gender	0.208	0.402	0.037		0.516		0.606		-0.585		1.000	0.690		1.450
Civil status	-0.682	0.415	-0.135		-1.643		0.102		-1.499		0.136	0.528		1.895
Children	-0.349	0.471	-0.068		-0.741		0.460		-1.276		0.579	0.421		2.376
Loss of a loved one	0.190	0.405	0.030		0.469		0.640		-0.608		0.988	0.873		1.145
Education	0.343	0.320	0.069		1.072		0.285		-0.287		0.972	0.854		1.171
Continuation of education	1.236	0.478	0.200		2.587		**0.010**		0.294		2.177	0.596		1.678
Distance from hostilities	0.327	0.273	0.080		1.196		0.233		-0.212		0.866	0.804		1.243
Place of residence	0.332	0.399	0.053		0.832		0.406		-0.455		1.119	0.867		1.153
Material status	0.047	0.327	0.009		0.143		0.887		-0.598		0.691	0.813		1.231
Health insurance	1.506	0.448	0.233		3.364		**0.001**		0.624		2.389	0.741		1.350
Savings	1.088	0.399	0.187		2.726		**0.007**		0.301		1.875	0.759		1.317
Material security	-0.390	0.346	-0.079		-1.128		0.261		-1.072		0.292	0.735		1.361

B – estimated regression coefficient; SE – standard error; beta - standardized regression coefficient; t- t-statistic (for testing coefficient); p - p-value (significance level); 95% CI − 95% confidence interval; tol. - tolerance (measure of multicollinearity); VIF - variance inflation factor (multicollinearity indicator)

## Discussion

Statistical analysis showed that physical QOL among participants in hostilities increased when respondents had savings, lived in the countryside, and resided farther from hostilities, whereas it decreased with age; scores on QOL in the physical health domain were significantly higher in women.

QOL in the psychological health domain was significantly higher in the individuals who had health insurance and savings. QOL in the social relationships domain increased with a subjective sense of material security (perceived economic stability) and decreased with age. QOL in the environmental domain increased when respondents continued education, had health insurance, and had savings.

### Savings

Savings can be interpreted as a resource that enhances an individual’s ability to maintain relative autonomy and control in wartime. Their importance was evident in three out of four areas of quality of life, namely physical health, mental health and the environment. The exception was the domain of social relations, where it was not the level of financial resources itself that proved more significant, but rather a subjective sense of material security. The three areas in which savings were significant are directly linked to individuals’ day-to-day functioning and their ability to meet basic life needs. In the context of physical health, this means, amongst other things, access to medicines, food and medical care. In the field of mental health, savings can increase the possibility of securing peace of mind, privacy and therapeutic support. In the environmental sphere, they offer a real opportunity to secure a safe place to live and to access energy, education, transport and local infrastructure. In this sense, savings function as an instrumental resource and a tool for survival, enabling the maintenance of relative autonomy and control in a situation of chronic crisis [47].

As opposed to the remaining domains, QOL in the domain of social relationships did not depend directly on respondents’ material resources but on their subjective sense of material security. The lack of financial resources does not necessarily mean a limitation in social functioning if the individual maintains an internal conviction about their ability to cope with the difficult situation [38]. The subjective sense of material stability seems to function as a filter that conditions readiness to form and maintain relationships regardless of the actual level of resources possessed. It is not the resource in itself but the way it is perceived that determines QOL in this domain.

### Health insurance

Health insurance turned out to be a significant predictor of QOL in two domains: psychological and environmental. In the case of psychological health, its significance can go beyond the function of access to medical services, since insurance can act as a buffer – a symbol of order, predictability, and protection in the situation of wartime threat. The very fact of having insurance can strengthen the sense of security and agency in the face of an uncertain situation, even if actual use of health care remains limited [20].

In the environmental domain, insurance can be understood as an indicator of the individual’s embeddedness in the institutional structure and their integration with the social system [9]. In the conditions of war, where institutions frequently fail or are inaccessible, insurance may mean relative stability and access to organized channels of assistance.

The lack of a significant relationship between having health insurance and QOL in the physical health domain suggests that what plays the decisive role in this area is directly accessible resources, such as savings or actual possibilities of using medical care, because in wartime conditions real access to health services frequently fails to coincide with the formal insurance status [18].

Likewise, in the social relationships domain of QOL, having health insurance was not significantly associated with the level of QOL, which may suggest that social ties are shaped by a subjective sense of security rather than by formal institutional guarantees [25].

### Age

Age turned out to be a significant predictor of QOL in only two domains: physical health and social relationships. In both cases, we observed a negative relationship: the higher the respondent’s age, the lower the QOL level. In the context of physical health, this result is intuitive, since with age there is an increase in the risk of somatic diseases and functional limitations, which can be particularly afflictive in wartime conditions [54]. What should also be considered is the issue of access to medical care. Older adults often have problems moving around, which can make it impossible for them to escape from dangerous zones and gain access to shelters, humanitarian aid, or food. This situation can lead to a deterioration in overall physical health. War interferes with the everyday structures of family and institutional care. Older adults who previously relied on support from their families or institutions (e.g., residential care homes) may be left with no one to care for them in the situation of war, which translates into a decline in their physical functioning.

A related aspect is the issue of QOL in the social relationships domain [46, 5, 33]. Namely, in the case of social relationships, the decline in QOL with age may stem from increasing isolation, deteriorating mobility, and the loss of members of one’s support network, which limits the possibilities of maintaining relations and actively participating in social life [45]. Moreover, older individuals may experience greater difficulties adapting to sudden social and infrastructural changes caused by war (e.g., evacuation, relocation, difficulties contacting their loved ones due to lack of access to technology).

### The remaining factors

In the analysis of QOL in the physical health domain, other significant predictors turned out to be place of residence and geographical distance from the zone of hostilities. Individuals residing in rural areas and those located farther from direct hostilities reported higher physical QOL. These results can be interpreted in terms of reduced exposure to war-related stressors. Rural areas are at a relatively lower risk of coming under fire and of infrastructure being destroyed, which translates into a higher sense of security and better access to natural resources [10]. Moreover, life in the countryside may be conducive to maintaining daily routine and self-sufficiency (e.g., regarding food and heating), which acquires special importance in wartime conditions. Greater distance from hostilities reduces the direct risk of violence, evacuation, or being left without a roof over one’s head, thereby reducing the somatic burdens that stem from chronic stress [40].

Importantly, women scored higher on QOL in the physical health domain, which indicates better QOL in this domain compared to men. Though this may seem paradoxical considering the care-related responsibilities and emotional strains that women face more often, there is data suggesting that in crisis situations women show greater adaptive flexibility, more often use social support systems, and more willingly engage in health behaviors (e.g., seeking medical help, taking care of hygiene, maintaining daily routine). In the literature it has also been pointed out that women have a higher level of what is referred to as health awareness [8], which may favor higher subjective evaluation of physical well-being compared to men [51].

In the case of environmental QOL, the significant predictor was continuation of education. This result can be interpreted as a sign of maintaining future-orientedness and the ability to engage in purposeful actions even in conditions of destabilization. Individuals who continue education, regardless of its level and form, not only maintain contact with institutions but also increase their chances of social and occupational integration [34]. In this context, therefore, education plays a function of structuring everyday life, giving a sense of direction, control, and belonging, which translates into higher environmental QOL. Additionally, education can increase access to information networks and local resources (e.g., scholarships, psychological support, student housing) [41], which are particularly valuable in wartime conditions. In conditions of destabilization, performing a specific social role, such as that of a participant in the education system, can therefore act protectively, offering a symbolic and practical point of support.

## Conclusions

The fact that the set of predictors varies across QOL domains makes it evident that QOL in wartime conditions is not a homogeneous category but a dynamically constructed dimension of existence, dependent on the character of needs and on the adaptation strategies adopted. While the physical health and environmental domains are conditioned to a greater extent by access to material and infrastructural resources (such as savings or distance from hostilities), in the domains of social relationships and psychological well-being it is subjective and systemic factors that acquire special significance. QOL in the social relationships domain was based on a subjective sense of material stability rather than actual financial resources, which suggests a different mechanism of their maintenance.

At the same time, continuation of education emerges as a significant predictor of QOL. This means that effective support strategies should go beyond the framework of interventions focused exclusively on securing elementary needs. It is essential that they include actions supporting agency, sense of direction, and the possibility of continuing purposeful and relational activities, which constitute the foundation of social immunity in conditions of prolonged crisis.

In other words, these results should encourage a redefinition of the framework of humanitarian support, from an approach focused exclusively on securing physical needs towards models including psychological flexibility, the possibility of continuing social roles, and access to the structures of social order, such as education and health insurance.

It is these elements that enable individuals to maintain a sense of meaning, agency, and relatedness, which are the foundation of immunity during a long-term crisis.

The practical implications of the findings relate primarily to the healthcare and social welfare systems in Ukraine. The WHOQOL-BREF may serve as a useful tool to assist in assessing the quality of life of internally displaced persons and identifying areas requiring health, psychological and social support. Its use may support the planning, monitoring and evaluation of aid measures aimed at those affected by the consequences of armed conflict.

With regard to countries hosting refugees from Ukraine, the results of this study should be regarded primarily as a reference point for further research and the planning of local relief efforts. Experience from Western European countries indicates that the WHOQOL-BREF can be used as a brief, multidimensional tool for assessing quality of life in both population-based studies and selected clinical contexts [4, 32, 39, 50]. However, its use outside Ukraine should take into account local organisational, linguistic and cultural conditions, as well as the specific characteristics of healthcare and social welfare systems [6, 43]. Furthermore, caution is required when interpreting the results in light of reports of psychometric differences between the language versions of the WHOQOL-BREF questionnaire [36, 49].

## Limitations and future research

Even though the study has yielded significant theoretical and practical findings, several limitations should be noted that may influence the interpretation of its results.

Firstly, the design of the study was cross-sectional, which does not allow for clear conclusions concerning the direction of causal relationships. In the future, it is advisable to conduct longitudinal research that would make it possible to trace the trajectory of changes in QOL over time.

Secondly, the structure of the sample, including mainly individuals from areas affected by the armed conflict and internally displaced ones, may limit the possibilities of generalizing the results to other populations in different cultural contexts. Further comparative studies are therefore recommended, including different geographical and situational groups.

Thirdly, however, it should be noted that the latest comparative analyses of the different language versions of the WHOQOL-BREF questionnaire indicate the presence of psychometric differences between the various translations, including the Ukrainian version, which may limit the comparability of results obtained across different populations and countries [36]. Consequently, the results obtained should be interpreted with caution, taking into account the potential influence of linguistic and cultural factors on the measurement properties of the tool used.

Fourthly, the regression models obtained were characterised by relatively low values of the adjusted R² coefficient, indicating that the variables included explained only a small proportion of the variance in the individual domains of quality of life. This result should be interpreted with caution. Quality of life is a multidimensional construct influenced by numerous biological, psychological, social and environmental factors not accounted for in this study. The aim of the analyses was to identify significant predictors of specific quality-of-life domains, rather than to develop models with high predictive power. Therefore, the results obtained should be interpreted as indicating the existence of significant relationships between the analysed variables, which require further verification in studies covering a wider range of potential determinants.

Finally, the measurement of QOL and loss of resources was based on self-report questionnaire-based tools, which carries a risk of self-report errors and contextual effects [35]. What should be considered in the future is the supplementing of quantitative data with qualitative components (e.g., interviews, case studies) that could deepen the understanding of adaptation mechanisms in wartime conditions.

## Data Availability

The datasets generated and/or analysed during the current study are not publicly available due to the sensitive nature of the data and the need to protect participant confidentiality, but are available from the corresponding author on reasonable request.
